# Supplementary material for: Cabs1 Maintains Structural Integrity of Mouse Sperm Flagella during Epididymal Transit of Sperm
Source: Int J Mol Sci. 2021 Jan 11;22(2):652. doi: 10.3390/ijms22020652 (PMC7827751; doi:10.3390/ijms22020652)
Supplement: Supplementary file 1 [file ijms-22-00652-s001.pdf]

Supplementary Materials:

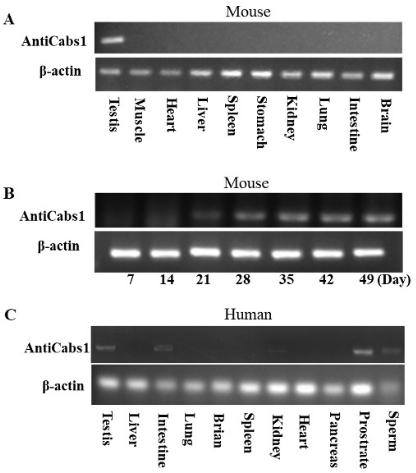

Figure S1. The expression profiles of *AntiCabs1* in mice and humans. (**A**) *AntiCabs1* exclusively expressed in mouse testis. (**B**) *AntiCabs1* expression in mouse testis at different stages of development. (**C**) *AntiCabs1* expression in human tissues. Total RNA was isolated from mouse tissue samples using a RNAiso Plus kit, and human tissue RNAs were purchased from Takara (Dalian, China). RT-PCR was performed to detect the *AntiCabs1* expression level. The *β-actin* gene was used as a loading control.

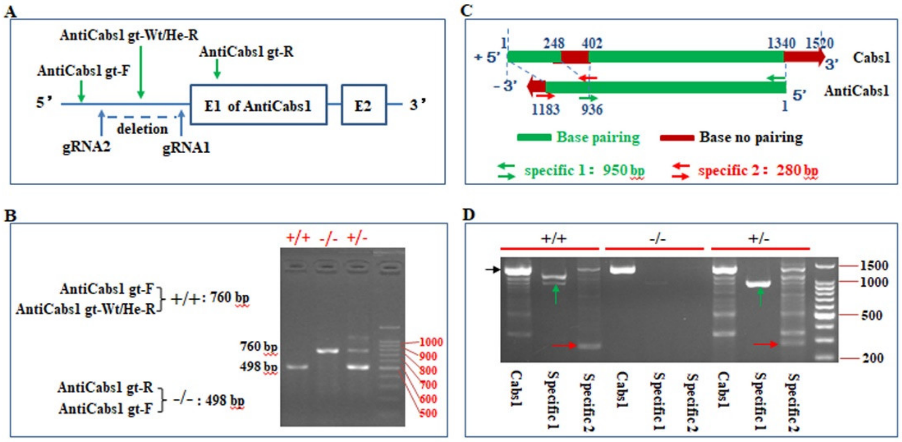

Figure S2. Generation of *AntiCabs1* KO mice. (**A**) The diagrams of the targeting strategy and genotyping primer sites. (**B**) Validation of the *AntiCabs1* KO mouse model. PCR genotyping was performed on tail genomic DNA with the two primer sets, allowing for the specific recognition of WT (760 bp) and KO (498 bp) alleles. (**C**) Schematic representation of *AntiCabs1*-specific RT-PCR primer. (**D**) Investigation of *AntiCabs1* expression in *AntiCabs1* KO mice. Total RNA extracts from the testis were used to confirm the absence of *AntiCabs1* transcripts (950 and 280 bp PCR products) by RT-PCR with specific two primer sets in KO mice.

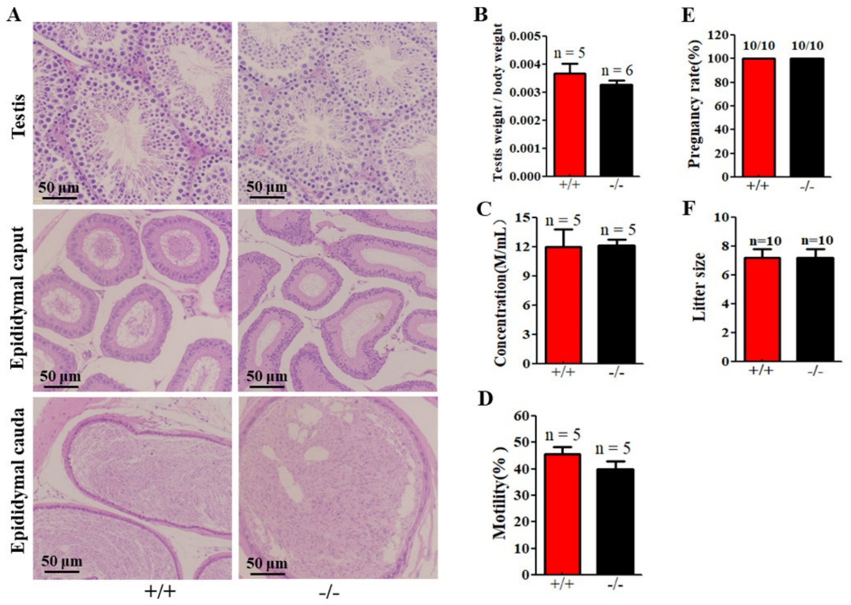

Figure S3. Testicular and epididymal phenotypes in *AntiCabs1* KO mice. (**A**) Histological analyses of the testis and epididymis sections from WT and *AntiCabs1* KO mice stained with hematoxylin and eosin. (**B**) Testis weight. The weight of the testis and body was weighed by an electronic balance. (**C**) Sperm concentration and (**D**) motility in male WT and KO mice were measured by computer-assisted sperm analysis (CASA) using the sperm collected from the cauda epididymitis. (**E**) Male fertility and (**F**) litter size. Pregnancy and litter size were counted in mating cages with male WT and KO mice over a period of 3 months.

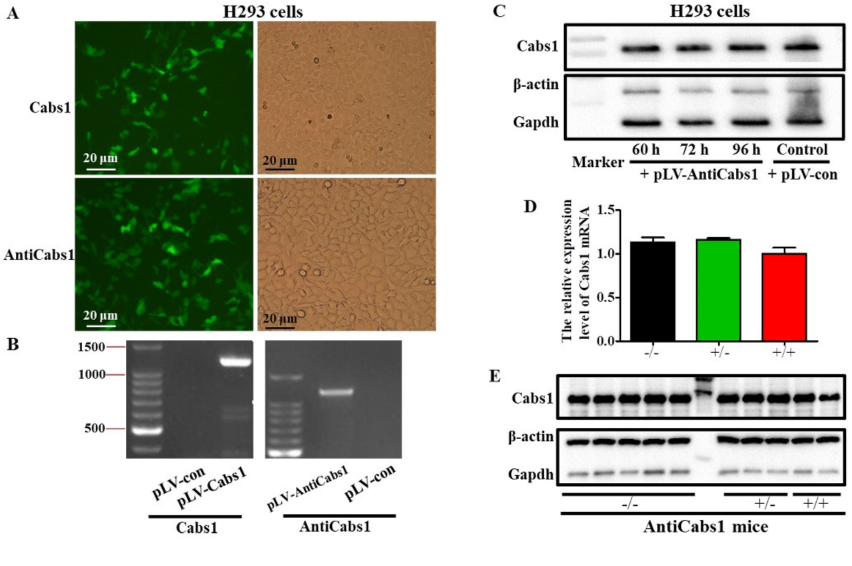

Figure S4. *Cabs1* expression is independent on *AntiCabs1*. (**A**) *Cabs1* and *AntiCabs1* overexpression of the H293 cell lines were constructed by transfecting the Enhanced Green Fluorescent Protein (EGFP)-labeled lentiviral vectors, pLV-Cabs1 and pLV-AntiCabs1, respectively. (**B**) Confirmation of *Cabs1* and *AntiCabs1* overexpression of the H293 cell lines. The cells that overexpressed *AntiCabs1* and *Cabs1* were used to isolate the total RNA, and then, RT-PCR was performed to amplify the *Cabs1* and *AntiCabs1* transcripts. (**C**) Effect of *AntiCabs1* on Cabs1 protein expression in vitro. Cabs1 expression levels were detected by Western blot after the Cabs1 overexpressed cells were treated with the *AntiCabs1* lentiviral vector for 60, 72, or 96 h. (**D**,**E**) Effect of *AntiCabs1* on *Cabs1* mRNA and protein expressions in vivo. *Cabs1* mRNA expression levels were detected by qPCR. Cabs1 protein expression levels were determined by Western blot in *AntiCabs1* homozygous, heterozygous, and WT mice. Gapdh and β-actin were used as loading controls.

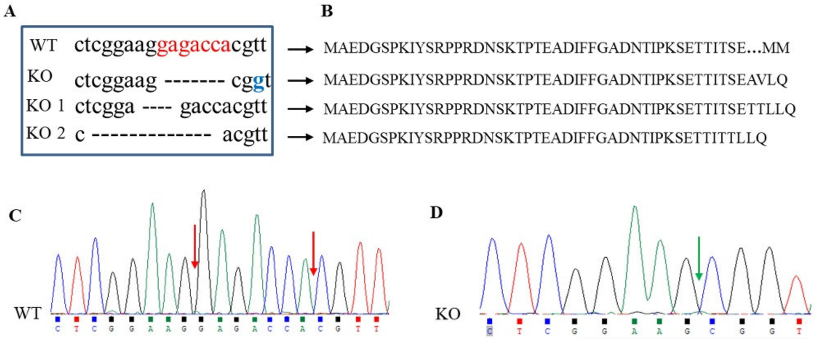

Figure S5. The generation and confirmation of *Cabs1* KO mice. (**A**) Three-line KO founder mice were produced. (**B**) Amino acid sequences encoded by wild-type and mutant *Cabs1*. (**C**,**D**) Sanger sequencing of *Cabs1* WT and KO alleles.

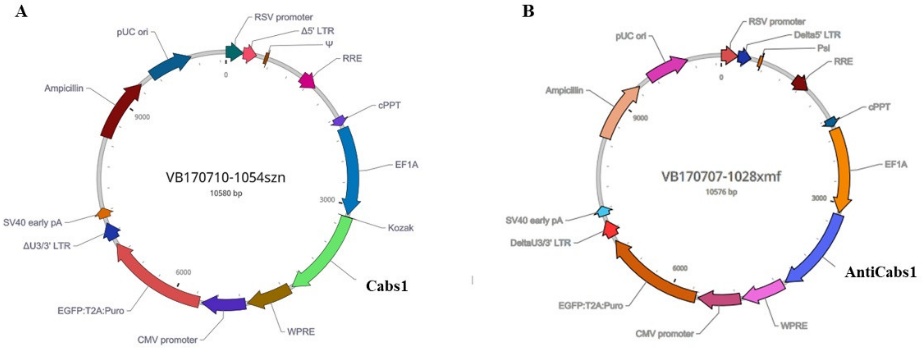

Figure S6. Schematic diagram of *Cabs1* (**A**) and *AntiCabs1* (**B**) lentivirus gene expression vectors. Vectors were constructed and packed by Cyagen US Inc. (Guangzhou, China).

Table S1. The primer sequences of the examined genes.

Purpose	Symbol/ID	Forward Primer	Reverse Primer	Length(bp)
Genotyping primers	Cabs1	TGGTGAGCCCCTGTCATTACCTG	AACAGCTCCATCATCAGGAGCATC	756
Sequencing Primers	Cabs1	CAATGCTTCAGTCACTATTCTGG		
qPCR	Cabs1	AGGTCACCACCATTCCAGACA	AGTGAGCAGAACAGCATGGG	257
PCR_CDS	Cabs1	ATGGCTGAAGATGGATCGC	TTACATCATGAGATCGTCTGGTTC	1176
Genotyping primers_KO	AntiCabs1	TGTGAACACCACAGATTTGCCTGA	AGTCTATTTACACCCACAC-TCCCTCTCA	760
Genotyping primers_WT	AntiCabs1	GAAATGGCTTCCTTTACTGCTT-GCC	AGTCTATTTACACCCACAC-TCCCTCTCA	498
qPCR	AntiCabs1	GGGATGAGGTGTGAGCTTGT	GGGCTGACAACACCATTCCTA	128
specific1_PCR	AntiCabs1	AATGCCCACAATATTCTTTTTTCT	AACAAGCTCACACCTCATCC	950
specific2_PCR	AntiCabs1	TGGGGATGAGGTGTGAGC	TTGCCCAGGGATGCCACT	280
PCR	AntiCabs1	AATGCCCACAATATTCTTTTTTCT	CTTGCCCAGGGATGCCACT	1208
qPCR	Ccnyl1	TGGACATTTTTGATGAGCGGT	ACCAACGTCACTATTGCACATTC	157
qPCR	Sept4	ACGGAATCGCAACAAACTGAC	TCTTTCTCCCGGATTAGCTTCTC	104
qPCR	Krt1	TGGGAGATTTTCAGGAGGAGG	GCCACACTCTTGGAGATGCTC	104
loading control	β-actin	GGCTGTATTCCCCTCCATCG	CCAGTTGGTAACAATGCCATGT	154
